# The Prostate Specific Membrane Antigen Regulates the Expression of IL-6 and CCL5 in Prostate Tumour Cells by Activating the MAPK Pathways^1^

**DOI:** 10.1371/journal.pone.0004608

**Published:** 2009-02-26

**Authors:** Marco Colombatti, Silvia Grasso, Alessandra Porzia, Giulio Fracasso, Maria Teresa Scupoli, Sara Cingarlini, Ornella Poffe, Hassan Y. Naim, Martin Heine, Giuseppe Tridente, Fabrizio Mainiero, Dunia Ramarli

**Affiliations:** 1 Department of Pathology, University of Verona, Verona, Italy; 2 Clinical Immunology, Giovanni Battista Rossi Hospital, Verona, Italy; 3 Department of Experimental Medicine, Institute Pasteur-Fondazione Cenci Bolognetti, University of Rome “La Sapienza”, Rome, Italy; 4 Interdepartmental Laboratory for Medical Research (LURM), University of Verona, Verona, Italy; 5 Department of Physiological Chemistry, School of Veterinary Medicine, Hannover, Germany; University of Birmingham, United Kingdom

## Abstract

The interleukin-6 (IL-6) and the chemokine CCL5 are implicated in the development and progression of several forms of tumours including that of the prostate. The expression of the prostate specific membrane antigen (PSMA) is augmented in high-grade and metastatic tumors. Observations of the clinical behaviour of prostate tumors suggest that the increased secretion of IL-6 and CCL5 and the higher expression of PSMA may be correlated. We hypothesized that PSMA could be endowed with signalling properties and that its stimulation might impact on the regulation of the gene expression of IL-6 and CCL5. We herein demonstrate that the cross-linking of cell surface PSMA with specific antibodies activates the small GTPases RAS and RAC1 and the MAPKs p38 and ERK1/2 in prostate carcinoma LNCaP cells. As downstream effects of the PSMA-fostered RAS-RAC1-MAPK pathway activation we observed a strong induction of NF-κB activation associated with an increased expression of IL-6 and CCL5 genes. Pharmacological blockade with specific inhibitors revealed that both p38 and ERK1/2 participate in the phenomenon, although a major role exerted by p38 was evident. Finally we demonstrate that IL-6 and CCL5 enhanced the proliferative potential of LNCaP cells synergistically and in a dose-dependent manner and that CCL5 functioned by receptor-mediated activation of the STAT5-Cyclin D1 pro-proliferative pathway. The novel functions attributable to PSMA which are described in the present report may have profound influence on the survival and proliferation of prostate tumor cells, accounting for the observation that PSMA overexpression in prostate cancer patients is related to a worse prognosis.

## Introduction

Prostate cancer is the most commonly diagnosed neoplasia in man in developed countries. Death from prostate cancer occurs largely in patients with the aggressive androgen-insensitive metastatic disease. A number of studies have recently demonstrated that a prominent role in tumor survival and progression can be attributed to soluble mediators present in the tumor microenvironment. Among these, Interleukin-6 (IL-6) has a fundamental role in the regulation of proliferation, apoptosis, angiogenesis and differentiation in many cell types and it is also implicated in the development and progression of several forms of tumours including that of the prostate [1], [2]. In fact, the expression of IL-6 and its receptor is consistently demonstrated in human prostate cancer cell lines and in freshly isolated cells from human prostate carcinoma and benign prostate hyperplasia [3], [4]. Clinically, the levels of IL-6 in serum are significantly elevated in many men with advanced, hormone-refractory prostate cancer [5], [6]. Further, IL-6 activates androgen receptor-mediated gene expression in LNCaP cells in vitro [7], [8], suggesting that IL-6 may play a critical role during the progression of prostate cancer. In addition, over-expression of IL-6 in androgen-responsive LNCaP cells promotes their androgen-independent growth in vitro and in vivo [9]. Recently, the chemochine CCL5 (RANTES) was found to be expressed by human prostate carcinoma cells and reported to stimulate their proliferation and invasion [10]. Thus, also CCL5 appears to be directly involved in the behaviour of prostate carcinoma cells. The gene expression of both IL-6 and CCL5 is mainly regulated at a transcriptional level, by the cooperative activity of NF-κB transcription factor with members of at least five different families of transactivators including AP-1 [1]. Noticeably, the cooperation between NF-κB and AP-1 appears to be essential for the constitutive deregulated production of IL-6 observed in the androgen-independent, aggressive prostate cancer cells [11]. Gene induction occurs depending on the ability of a variety of cell surface receptors to activate distinct and/or partially overlapping intracellular signalling pathways eventually targeting the phosphorylation site(s) of one or more MAP kinases (i.e. p38, ERK1/2, JUNK) committed in turn to activate IL-6 and/or CCL5 gene transactivators. Cytokines, growth factors receptors, adhesion molecules and many other membrane-generated signals all share the ability to efficiently promote IL-6 or CCL5 gene expression and consequently also their downstream effects. In addition, under long-term treatment conditions, IL-6 can activate its own gene expression and, in prostate cancer, autocrine and paracrine loops involving IL-6 and one of its multiple activators, the TGF-beta, have been implicated in the regulation of cell proliferation, survival, and neuroendocrine differentiation [12].

The expression levels of the prostate specific membrane antigen (PSMA) have been proposed as a useful indicator of the severity of the disease in prostate cancer [13]–[15]. PSMA is a type-II integral membrane protein, predominantly localized to the epithelial cells of the prostate gland and endowed with folate-hydrolase and carboxypeptidase activity [13]. Its low expression in normal prostate epithelial cells increases several fold in high-grade prostate cancers, in metastatic and in androgen-insensitive prostate carcinoma [14]. These features have made it emerging as one of the most promising biomarkers in the diagnosis and treatment of prostate cancer [14], [16]. The clinical observations suggesting a possible correlation between high levels of IL-6 production and PSMA expression in high-grade prostate cancer prompted us to investigate whether a functional relationship may exist between the presence of PSMA at the cell surface and the level of gene expression of IL-6. CCL5 was also investigated because it shares with IL-6 the mechanism of gene induction and the pro-proliferative activity. The hypothesis that PSMA belonged to the ever increasing category of molecules endowed with signalling properties was also suggested by previous observations showing that silencing, inhibiting or knocking-down PSMA modulates the adhesion/de-adhesion processes via activation of focal adhesion kinase (FAK) in LNCaP cells or via activation of p21-activated kinase1 (PAK1) in normal endothelial cells (HUVEC) [17], [18]. Unlike what previously done by others [18], [19], we decided to stimulate rather than to inhibit or silence PSMA at the surface of LNCaP cells and to overcome the lack of defined PSMA ligand(s) by cross-linking the extra-cellular domain of the molecule with specific antibodies.

Here, by using the androgen-independent LNCaP cell line, we present for the first time evidence that the antibody-mediated aggregation of PSMA greatly induce the basal gene expression of both IL-6 and CCL5 in LNCaP cells. Gene induction occurs due to an activation cascade involving RAS, RAC1, p38 and ERK1/2 MAPKs, leading to the phosphorylation of the p65 subunit of NF-κB transcription factor. In addition, we demonstrate that both IL-6 and CCL5 promote the proliferation of LNCaP tumour cells reaching their maximal activity synergistically. Moreover, we present a novel finding related to the ability of CCL5 to induced proliferation in LNCaP cells thank to the activation of STAT5 and the consequent increase of expression of cyclin D1, a pathway controlling cell growth in normal and prostate cancer cells. This newly discovered functions of PSMA implicate this molecule as an important regulator of the prostate tumour cell growth.

## Materials and Methods

### Chemicals, cell culture reagents and antibodies

PD098059 and SB202190 inhibitors were from Calbiochem-Novabiochem (San Diego, CA). Poly-D-lysine, trypsin and protease inhibitors were from SIGMA (SIGMA, Milan, Itay), whereas phenylmethylsulfonyl fluoride, antipain and soybean trypsin inhibitor were from Roche Diagnostics (Mannheim, Germany). The Hybond-P PVDF membranes and the ECL plus Western Blotting Detection System were from Amersham Bioscience Inc. (Freiburg, Germany). RPMI 1640 medium and glutamine were from Società Prodotti Antibiotici (Milan, Italy). Fetal bovine serum (FBS) was from Celbio (Pero, Milan, Italy).(Fab)2 goat anti-mouse Ig and anti-VCAM-1 were from Immunotech (Marseille, France); FITC-labeled (Fab)2 goat anti-mouse Ig and anti p65 phosphorylated mAb were from Becton-Dickinson (San Josè, CA); the anti PSMA mAb J591, described by H. Liu et al. [20] was kindly supplied by Dr. N.H. Bander (Medical College of Cornell University, New York); the 7E11c mAb, recognizing a cytosolic epitope of PSMA was purified from the supernatant of the hybridoma HB-10494 (ATCC, American Type Culture Collection, Rockville Pike, MD); anti-phospho-p38, anti-p38 polyclonal Abs and anti-phospho-ERK1/2 mAb were from Cell Signaling Technology (Danvers, MA); anti-STAT 5 and anti-cyclin D1 polyclonal antibodies were from Santa Cruz Biotechnology (Santa Cruz, CA); anti-RAS (clone RAS10) and anti-RAC (clone 23A8) mAbs were from Upstate Biotechnology (Lake Placid, NY). The anti-PSMA mAb used for this work was produced in our laboratory and used in purified form following affinity chromatography. Briefly, BALB/c mice were immunized with a soluble recombinant form of PSMA and hybridoma cells obtained following standard fusion procedures. Hybrids were screened by flow cytometry using PSMA positive (LNCaP) and PSMA negative (DU145, PC-3) cell lines.

### Cells and cell treatments

The PSMA-positive LNCaP and the PSMA-negative DU145 and PC-3 human prostate carcinoma cell lines were from ATCC. Cells were maintained in vitro by serially passaging in RPMI-10% FBS at 37°C in a humidified atmosphere of 5% CO_2_. In the case of the LNCaP cell line, plating was performed on poly-D-lysine coated tissue culture plasticware (10 µg/ml). When required, cells grown to confluence in 24 well plates were incubated with saturating doses of the appropriate mAb (5 µg/ml, 30 min at room temperature), washed and placed at 37°C for different times with (Fab)_2_-goat anti-mouse (Gam) (10 µg/ml) to induce the clustering of PSMA molecules at the cell surface (this procedure will be henceforth designated “PSMA cross-linking”). In some instances, cells were pre-treated (6 h at 37°C) with ERK1/2 (PD 98059) or p38 (SB 202190) inhibitors, used alone or in combination, at 25 or 50 µM in complete medium. The inhibitors were present throughout the duration of the assays. Cell-free supernatants and cells were separately recovered from each sample of untreated or treated cells 24 h after the addition of the various compounds. The volume of each cell-free supernatant was measured and the supernatant used to determine IL-6 and CCL5 production. Cells corresponding to each supernatant were lysed and the amount of proteins assessed by Coomassie protein assay reagent (Pierce, Celbio, Milano, Italy).

### Biochemical methods

Cell extracts were prepared from LNCaP cells cultured overnight with 2.5% FBS and then starved from serum for 2 h. Protein amount was measured by Coomassie protein assay reagent. RAS activity was assessed by employing the RAS activation assay kit (Upstate Biotechnology). Briefly, untreated and treated cells were lysed for 30 min on ice in a buffer containing 25 mM Hepes pH 7.5, NaCl 150 mM, 10% Glicerol, Na orthovanadate 1 mM, Na pyrophosphate 50 nM, Na fluoride 25 mM, MgCl2 10 mM, 1% NP40, phosphatase and protease inhibitors. The cell lysates (250 µg) were incubated with 10 µl of glutathione S-transferase (GST)-Raf-RAS binding domain (BD) (Upstate Biotechnology) coupled to agarose for 1 h at 4°C. The beads were washed with the lysis buffer, resuspended in 2× SDS sample buffer, and separated by SDS-Page. Western Blot was performed with the anti-RAS mAb and immunoreactive bands were visualized by using a horseradish peroxidase-conjugated secondary antibody and the ECL system (GE Healthcare Bio-Sciences AB, Uppsala, Sweden).

RAC1 activity was assessed by employing the RAC activation assay kit (Upstate Biotechnology). Briefly, cell lysates prepared as above (250 µg) were incubated with 10 µl of GST-PAK-1-RAC BD coupled to agarose for 1 h at 4°C. The beads were washed with the lysis buffer, resuspended in 2× SDS sample buffer and separated by SDS-Page. Western blotting was performed with the anti-RAC antibody (Upstate Biotechnology).

To examine p38 and ERK1/2 phosphorylation, untreated and treated cells were extracted with the buffer indicated above. Equal amounts of total proteins were boiled in sample buffer and separated by SDS-PAGE. Following immunoblotting with anti-phospho-p38 or anti-p38 polyclonal Ab, anti-phospho-ERK1/2 mAb or anti-ERK1/2 polyclonal Ab, immunoreactive bands were visualized by using horseradish peroxidase-conjugated secondary antibody and ECL. Film densitometry was carried out with a laser densitometer and analyzed using a built-in software (LKB, Bromma, Sweden).

### Intracellular phospho-specific flow cytometry

LNCaP cells were treated with ant-PSMA mAb for 30 min at room temperature, washed and cross-linked with Gam at 37°C for 0, 15 or 30 min. After this time cells were fixed with Fix buffer (BD) for 10 min at room temperature to arrest signaling activity. Cells were then washed and permeabilized with 50% ice-cold methanol for 30 min on ice. Fixed and permeabilized cells were washed twice, resuspended in phosphate-buffered saline plus 2% FBS and stained for 30 min at room temperature with monoclonal PE-conjugated antibody against phospho-NF-κB (S529) (Becton-Dickinson). Conjugated isotype-matched, non-reactive monoclonal antibody was used as control. Fluorescence signals were detected using a FACSCalibur flow cytometer (Becton-Dickinson) and data elaborated by FlowJo software (Tree Star, Ashland, OR).

### IL-6 and CCL5 quantitation

IL-6 and CCL5 concentration in cell-free supernatants was measured with an ELISA kit used according to the manufacturer's instructions (Endogen, Woburn, MA). Optical density (OD) values were plotted onto a standard curve and expressed as pg/ml. Having measured the volume of cell-free supernatants of each sample, IL-6 or CCL5 values could be re-expressed as total IL-6 or CCL5/sample. Total IL-6 or CCL5 of each sample was then normalized versus the amount of total protein of cell lysate of the producing cells and calculated as pg IL-6 or CCL5/ mg of protein lysate.

### Cell proliferation assays

Cell proliferation studies were carried out with LNCaP cells previously cultured with only 1% FBS for 48 h and then treated with the indicated doses of recombinant IL-6 or CCL5 (Endogen). In the case of treatment with anti-PSMA mAb the binding and the cross-linking of anti-PSMA or 7E11c were performed as previously described in the “Cells and cell treatments” paragraph of the present section. In the case of anti-CCR5 treatment, the antibody at the indicated concentrations was preincubated for 2 h at 37°C before adding the CCL5. During the last 12 h cells were pulsed with 1 µCi[methyl-^3^H] thymidine (^3^H-TdR) (6.7 Ci/mmol) from NEN Dupont (Boston, MA). Cells were harvested onto glass-fiber filters and radioactivity measured in a beta-spectrometer. Results were expressed as the percentage of incorporation of the untreated control.

### Flow cytometry

Cells were incubated with saturating amounts of the indicated antibody for 1 h on ice and then washed three times with cold RPMI medium. The cells were then incubated with 2 µl of a goat anti-mouse FITC-labeled antiserum (Becton-Dickinson) for 1 h on ice. After washings cells were analyzed by a Facs Canto instrument (Becton-Dickinson).

### Statistical analysis

Statistical analysis of results was performed applying the parametric unpaired t test. Significance was accepted when p<0.05.

## Results

### Characteristics of the anti-PSMA mAb used for the present study

**Figure 1:**
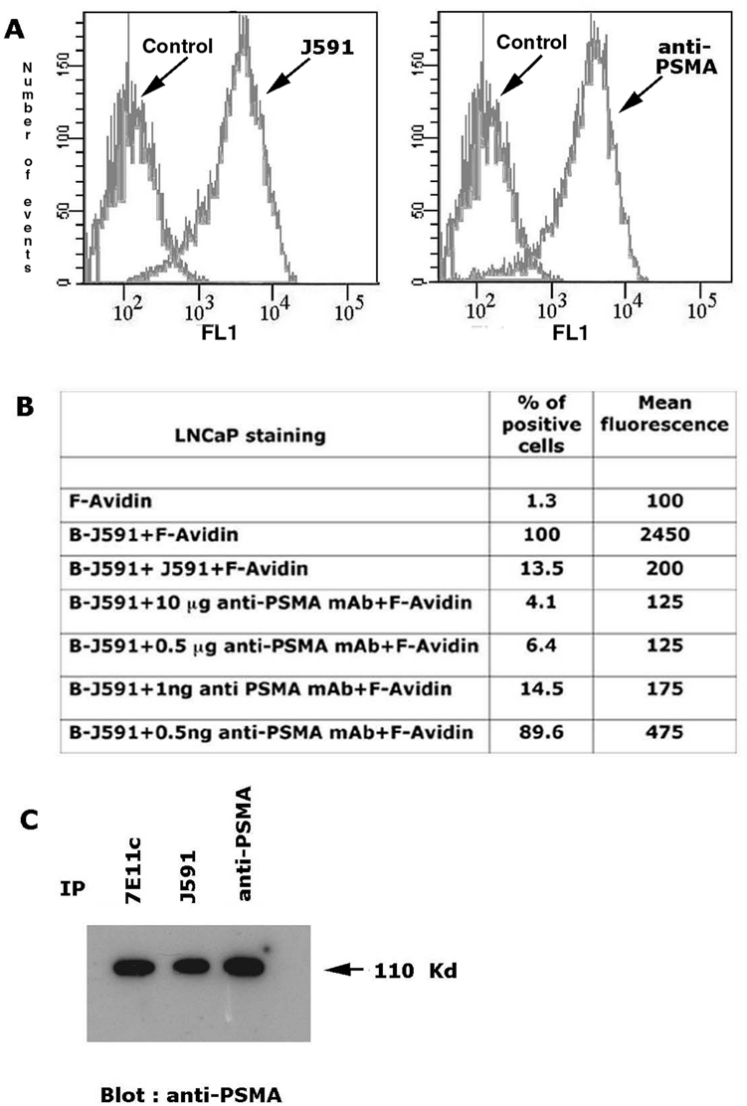
Reactivity of anti-PSMA mAb. Panel A: representative flow cytometry of LNCaP cells stained with anti-PSMA or with J591 mAb. Controls are represented by cells goat anti-mouse-FITC IgG only. Panel B: flow cytometry of LNCaP cells incubated with biotinylated J591 mAb (B-J591) with or without pretreatment (45 min on ice) with anti-PSMA at the indicated concentrations or with non-biotinylated J591 mAb as control. Antibody binding was revealed by FITC-Avidin (F-Avidin). The percentage of positive cells and the Mean Fluorescence Intensity observed under the different conditions are representative of two independent experiments performed with independent cultures. Panel C: Western blot analysis of LNCaP cell lysates immuneprecipitated with the indicated mAbs, and then probed with the anti-PSMA mAb followed by a goat anti-mouse antiserum labelled with horseradish peroxidase. The apparent molecular weight of PSMA band is indicated by the arrow.

Biochemical and functional studies shown in this paper were performed with an anti PSMA mAb (henceforth designated “anti-PSMA mAb”) produced by our group. Specificity for PSMA is shown in Fig. 1. Staining, competition and immuneprecipitation experiments were performed with LNCaP cells and the anti-PSMA mAb was compared with the J591 mAb, a reference mAb recognizing an extra-cellular epitope of the PSMA molecule [20]. Flow cytometry of cells stained with anti-PSMA or J591 mAbs demonstrated that both mAbs recognized a surface molecule expressed at comparable levels on LNCaP cells (Fig. 1A). No staining was instead observed with the PSMA negative DU145 or PC3 cell lines (not shown). Competition experiments, performed with biotinilated J591 mAb revealed by FITC-streptavidin, demonstrated that the number of positive cells and the mean fluorescence observed following J591 staining was progressively decreased when cells were pre-treated with increasing amounts of non-conjugated anti-PSMA mAb (Fig. 1, B), thus demonstrating that our anti-PSMA mAb specifically recognizes an epitope of the extra-cellular domain of the molecule identical or spatially close to that identified by the J591 mAb. Immunoprecipitations of crude cell lysates revealed the ability of our anti-PSMA mAb to immunoprecipitate a protein of the same apparent molecular weight (110 kD) of that immunoprecipitated using J591 mAb or 7E11c mAb, this last recognizing an intracellular epitope of PSMA (Fig. 1C). Collectively, these results demonstrated that our anti-PSMA mAb specifically recognizes an extra cellular epitope of PSMA. Results obtained at a single cell level were extended by immunohistochemistry to human tissues, demonstrating that the reactivity of anti-PSMA mAb overlapped that of J591 (not shown)

### PSMA recruitment with specific antibodies activates the MAPKs p38 and ERK1/2 and the small GTPases RAC1 and RAS in LNCaP cells

**Figure 2:**
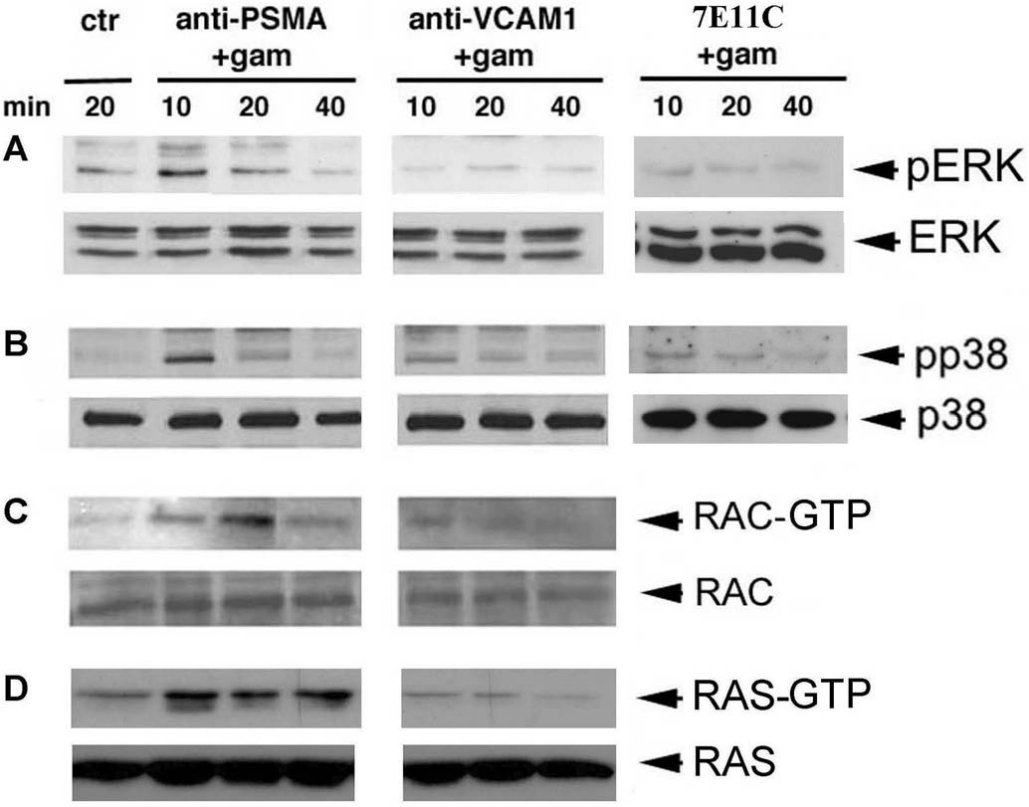
Kinetics of ERK1/21/2, p38, RAC 1 and RAS phosphorylation induced by PSMA cross-linking. LNCaP cells were left untreated or subjected to PSMA cross-linking for the indicated times at 37°C. An anti-VCAM-1 mAb was used as the isotype matched control. Panels A and B: p38 and ERK1/2 activation was assessed in crude lysates. Equal amounts (20 µg) of total proteins were boiled in sample buffer and separated by SDS-PAGE. Following immunoblotting with an anti-phospho-p38 or anti-phospho-ERK1/2 mAb, immunoreactive bands were visualized by using horseradish peroxidase-conjugated secondary antibody and the ECL system. Panels C and D: RAC1 and RAS activation was evaluated by a specific assay as described in the Material and Methods section. Bound active GTP-RAC and GTP-RAS molecules were analyzed by Western blotting using an anti-RAC1 or anti-Ras mAb and visualized as above. Total amount of ERK1/2, p38, RAC1 and RAS in crude lysates are shown as loading control at the bottom of each gel. Results are representative of one of three independent experiments.

To test whether PSMA was able to transduce intracellular signals leading to p38 and/or ERK1/2 phosphorylation, LNCaP cells were subjected to PSMA cross-linking by anti-PSMA-specific antibodies as adherent, confluent monolayers. PSMA molecules, recognized by the anti-PSMA mAb were induced to cluster at the cell membrane due to the binding of a goat anti-mouse IgG antiserum (Gam) added to cell cultures at 37°C for the indicated times. Controls included treatment with anti VCAM-1, with 7E11c or with Gam alone and the use of untreated cells. Cells were then washed and used as a source of cell lysates. We first assayed the phosphorylation of p38 and ERK1/2 in crude lysates obtained from the same cell cultures immunoblotted with antibodies specifically detecting the active, phosphorylated form of the two kinases. As shown in Fig. 2A and B, ERK1/2 and p38 were found activated at low levels in untreated LNCaP cells. Upon PSMA cross-linking, both ERK1/2 and p38 became considerably phosphorylated at 10 min. Sustained levels of phosphorylation lasted up to 20 min, and then returned to basal levels. It is known that the small GTPases RAS and RAC1 lie upstream on the MAPKs activation pathways [21]. Thus, to examine if PSMA cross-linking could activate also RAC1 in LNCaP cells, we performed an activation assay with a GST-Pak fusion protein which is able to bind and precipitate the active form of RAC1. As shown in Fig. 2C, PSMA cross-linking determined endogenous RAC1 activation in the same lysates. Similar results were obtained in the case of RAS, whose constitutive activation level was greatly augmented upon PSMA cross-linking (Fig. 2D). Cross-linking performed with an isotype matched mAb recognizing VCAM-1 failed to induce activation in all assays and at all points of the time-course (Fig. 2, all panels). Gel densitometry of independent experiments performed with different cell lysates confirmed the significance of the increased activation of ERK1/2, p38, RAC1 and RAS observed following PSMA, but not VCAM-1 cross-linking (Table 1). Because the time-course of RAS and RAC1 activation paralleled that of p38 and ERK1/2, these results overall indicate that PSMA is able to induce a sustained activation of the RAS- and RAC-MAPK pathways.

**Table 1:** Kinetics of ERK1/2, p38, RAC1 and RAS activation in LNCaP cells following cross-linking of anti-PSMA, anti-VCAM-1 or 7E11c mAba).

Protein	No treatment	Anti-PSMA cross-linking			Anti VCAM-1 cross-linking			7E11c cross-linking		
		10 min	20 min	40 min	10 min	20 min	40 min	10 min	20 min	40 min
ERK1/2	23±6.6b)	48±4.4 (p<0.05)c)	30±1.2 (p<0.05)	9.2±3.9 (ns)d)	8.4±3 (ns)	11±2.9 (ns)	9.3±2 (ns)	8.6±3.1 (ns)	9.8±4.7 (ns)	7.4±1.3 (ns)
P38	2.6±0.16	30.1±1.2 (p<0.001)	11.6±0.9 (p<0.001)	4.2±0.9 (ns)	5.4±2.2 (ns)	3.7±0.2 (ns)	3±0.7 (ns)	4.8±2.8 (ns)	3.2±3.4 (ns)	4.5±2.1 (ns)
RAC1	17.2±2.1	27.6±2.2 (p<0.05)	40.3±3.9 (p<0.01)	11±2.9 (ns)	29±8.8 (ns)	27±4.2 (ns)	19.2±3.8 (ns)	nde)	nd	nd
RAS	6.4±2.5	23.5±2.8 (p<0.001)	23.1±1.9 (p<0.001)	4.8±3.8 (ns)	13.2±3.6 (ns)	8.4±5 (ns)	20.5±9 (p<0.01)	nd	nd	nd

a) The 7E11c mAb recognizes an intracellular PSMA epitope and is therefore used as a further control of the experiment described.

b) Results of gel densitometry are expressed as the ratio±SEM of activated/total protein as evaluated by measuring the respective areas.

c) Statistical significance is reported with respect to the “No treatment” data.

d) ns = not significant.

e) nd = not determined.

### PSMA-elicited phosphorylation of p38 and ERK1/2 induces the activation of NF-κB transcription factor

**Figure 3:**
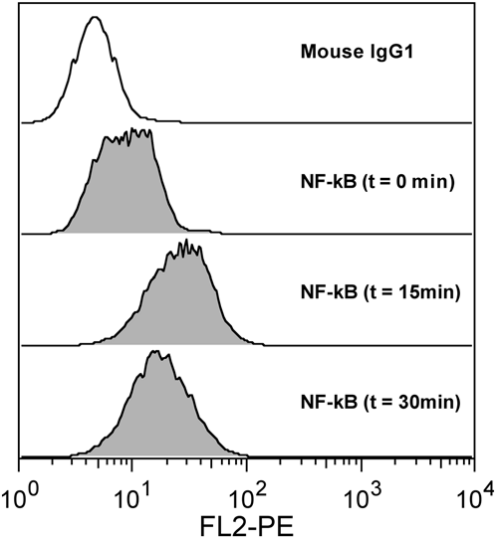
NF-κB activation induced by PSMA crosslinking. LNCaP cells were subjected to stimulation by cross-linking of PSMA or of VCAM-1 for 45 min at room temperature and then cultured for the indicated times at 37°C. The cells were then harvested, washed, permeabilized and stained with a PE-labelled anti-phosphorylated NF-κB p65 antibody. Cells were then analyzed by flow cytometry. The MFI was recorded. An isotype-matched IgG1 antibody was used to mock-treat LNCaP cells; the Figure illustrates a representative experiment out of three performed independently. MFI values of controls were: untreated cells 9.22±0.9, cells treated with an anti-VCAM-1 mAb 5.74+0.09, cells treated with a mouse IgG1 mAb 5.02±0.3. No substantial differences in the control samples were observed at the various time points of the experiments. MFI of Anti-PSMA cross-linked cells were: 22.03±2.2 and 15.6±1.3 at 15 min and 30 min, respectively. p values of samples treated with anti-PSMA mAb were <0.01 at 15 min and 30 min, respectively, with respect to the untreated cells. The first panel from top (mouse IgG1) is the plot corresponding to time 0.

It is well established that p38 and ERK1/2 have a role in the process of activation of NF-κB transcription factor, and that NF-κB plays a major function in the transcriptional control of various cytokines and chemokines, including IL-6 and CCL5 [1]. To investigate whether NF-κB activation was induced in LNCaP cells by the cross-linking of PSMA, cells were treated as described in Fig. 2 and stained with a mAb recognizing the phosphorylated Serine 529 (pS529) located in the transactivation domain of the p65 subunit of the human NF-κB. As the pS529 containing domain is unmasked only after detachment of NF-κB inhibitors induced by stimulation, the exposure of pS529 is considered a marker of NF-κB nuclear translocation and activation in normal and neoplastic cells of different origin. Additionally, phosphorylated S529 further augments NF-κB transactivation potential [22]. Controls were represented by cells left untreated, treated with an isotype-matched IgG1 antibody or subjected to VCAM-1 cross-linking. The results of flow cytometry analysis (Fig. 3) demonstrated that the mean fluorescence intensity (MFI) of LNCaP cells (9.2±0.9 SEM) was greatly augmented following PSMA cross-linking, peaking at 15 min from stimulation and declining 30 min later (230%±30 SEM and 160%±20 SEM of control cells, respectively), thus implying that NF-κB activation had occurred. No increase of MFI was instead detected at the various points of the time course in the presence of the control antibody or VCAM-1 cross-linking.

### PSMA cross-linking enhances the production of IL-6 and CCL5 in LNCaP cells in a p38 and ERK1/2 dependent manner

**Figure 4:**
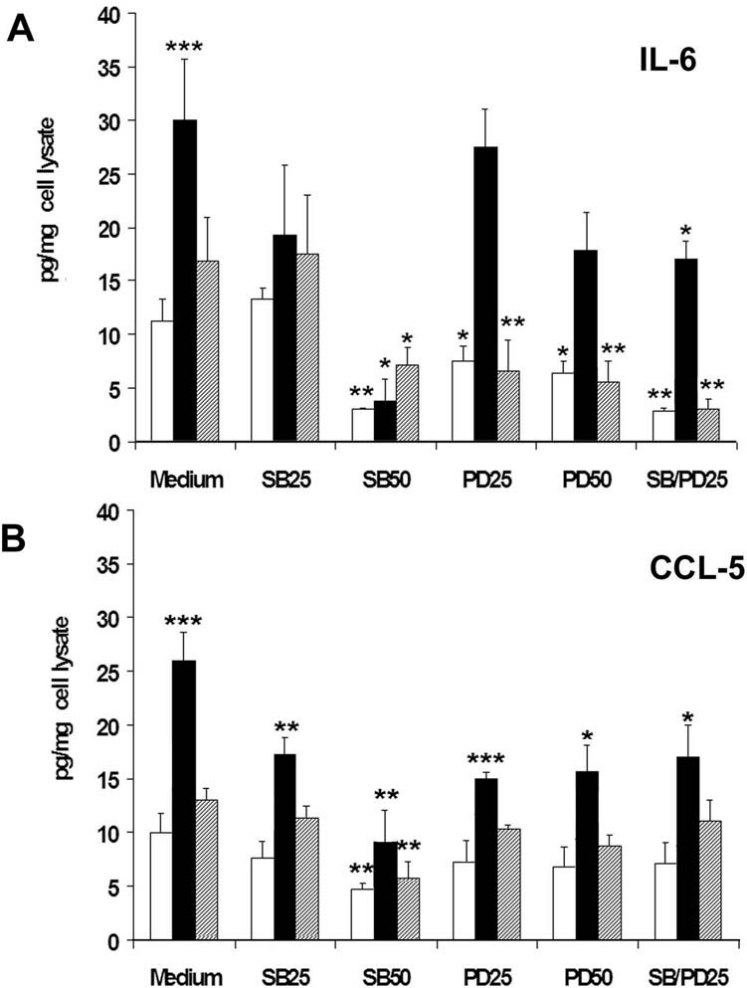
Inducing activity of PSMA cross-linking on IL-6 and CCL5 expression and role of PSMA-activated p38 and ERK1/2 . IL-6 (panel A) and CCL5 (panel B): production by LNCaP cells left untreated (open bars), stimulated by cross-linking via anti PSMA mAb (filled bars) or anti-VCAM-1 mAb (dashed bars) in the presence or in the absence of single or combined inhibitors (SB and PD) at the indicated doses (SB50 = 50 µg/ml, SB25 = 25 µg/ml; PD50 = 50 µg/ml, PD25 = 25 µg/ml; SB/PD25 = combinations of SB+PD at 25 µg/ml each). Control and treated cell cultures were washed, cultured for further 24 h before collecting cells and cell-free supernatants. IL-6 and CCL5 accumulation in each supernatant was measured by ELISA and expressed as pg/mg of cell lysates/24 h. Inhibitors were added to the cells 6 h prior to stimulation and maintained throughout the duration of the experiment. Values represent the mean±SEM of results of four experiments performed in duplicates. Stars on top of the bars indicate statistical significance as follows: p<0.001, ***; p<0.01, **; p<0.05, *.

Results shown in Fig. 2 and 3 demonstrated that cross-linking of PSMA recruited a signalling pathway eventually leading to NF-κB activation and nuclear translocation. The contribution of PSMA to the regulation of the production of IL-6 and CCL5 in LNCaP cells was then investigated in cross-linking experiments carried out as in Fig. 2. Further, we analyzed the role of PSMA-activated p38 and ERK1/2 in a loss-of-function condition achieved by using two pharmacological inhibitors: SB 202190 (SB), a compound binding specifically p38 and blocking reversibly its enzymatic activity or PD 98059 (PD), an inhibitor specifically preventing the activation of the ERK1/2-activating kinase MEK-1 [23]. LNCaP cells were stimulated as confluent monolayer and harvested 24 h later. To determine the basal and inducible release of IL-6 and CCL5 in culture, LNCaP cells and the corresponding cell-free supernatants were separately recovered from each treated or untreated cell culture. Cell lysates and cell-free supernatants were used to determine respectively the total protein amount and IL-6 and CCL5 accumulation in the supernatant of each sample. The results of IL-6 and CCL5 measurements were then expressed as pg of IL-6 or CCL5/mg of cell protein lysates. As illustrated in Fig. 4 A, LNCaP cells produced low amounts of IL-6 and of CCL5 basally (11.3±2 and14±3.6 pg/mg total cell protein/24 h, respectively). The cross-linking of PSMA increased this production by three-fold in the case of IL-6 and by two-fold in the case of CCL5 (311%±48, p<0.001 and 214%±38 p<0.001, respectively), thus demonstrating that PSMA recruitment at the surface of LNCaP cells powerfully up-regulates the production of both IL-6 and CCL5 at the same time. The cross-linking of the isotype-matched anti VCAM-1 mAb lacked instead any significant activity (155%±52 and 133%±11, respectively).

As also illustrated in Fig. 4 A, the p38 inhibitor SB used at 50 µM concentration almost abrogated both the basal and the PSMA-induced IL-6 production (70%±2.5 p<0.01 and 87.4%±20 p<0.05, respectively), whereas at 25 µM the decrease observed did not reach significance. The ERK1/2 inhibitor PD showed a poorer activity than SB, as it diminished the IL-6 produced basally, (30%±5 p<0.05 and 50%±7 p<0.05, at 25 µM and 50 µM, respectively) but failed to show any effect on that induced by PSMA cross-linking. Accordingly, IL-6 reduction observed in the presence of both inhibitors (both used at 25 µM concentration) overlapped that dependent on the sole SB. Similar results were obtained regarding the production of CCL5. As shown in Fig. 4 B, the basal production of CCL5 diminished in a dose-dependent manner in the presence of SB, reaching significance at 50 µM (45%±12, p<0.01). The PSMA-induced production of CCL5 was significantly reduced already at 25 µM SB and flattened out to basal levels at 50 µM (38%±4,p<0.01 and 72%±16, p<0.01, respectively). Also in the case of CCL5 the ERK1/2 inhibitor PD performed less efficiently. The basal production of CCL5 was not significantly affected by the presence of PD and the PSMA-induced CCL5 production was only halved although the effect was significant (p<0.001), with no dose-dependency (49%±4, p<0.001 PD used at 50 µM; 42±12, p<0.01, PD used at 25 µM). No greater activity was obtained by using a mixture of the two inhibitors used at 25 µM concentration. The possibility that the mix of inhibitors used at higher concentrations may be effective could not be explored because the compounds precipitate when the concentration is brought close to 100 µM. The effect of SB and PD on IL-6 and CCL5 produced by cells stimulated by the cross-linking of the control mAb were similar to those of untreated cells (Fig. 4 A and B).

Collectively, the results shown in Fig. 4 demonstrate that the activation of p38 and ERK1/2 elicited by PSMA cross-linking regulates the expression of IL-6 and CCL5 genes in LNCaP cells like in other pathological and normal epithelial cells [24], [25]. Moreover, as also observed in other instances our results underline the fact that p38 exerts a wider and greater activity than ERK1/2 on IL-6 and CCL5 gene expression [24], [25].

### Proliferation of LNCaP cells is inducible by IL- 6, CCL5 or by cross-linking of PSMA

Proliferation assays were then carried out to evaluate whether the increased availability of IL-6 and CCL5 in a neoplastic microenvironment would affect the growth of the LNCaP tumour cell line.

**Figure 5:**
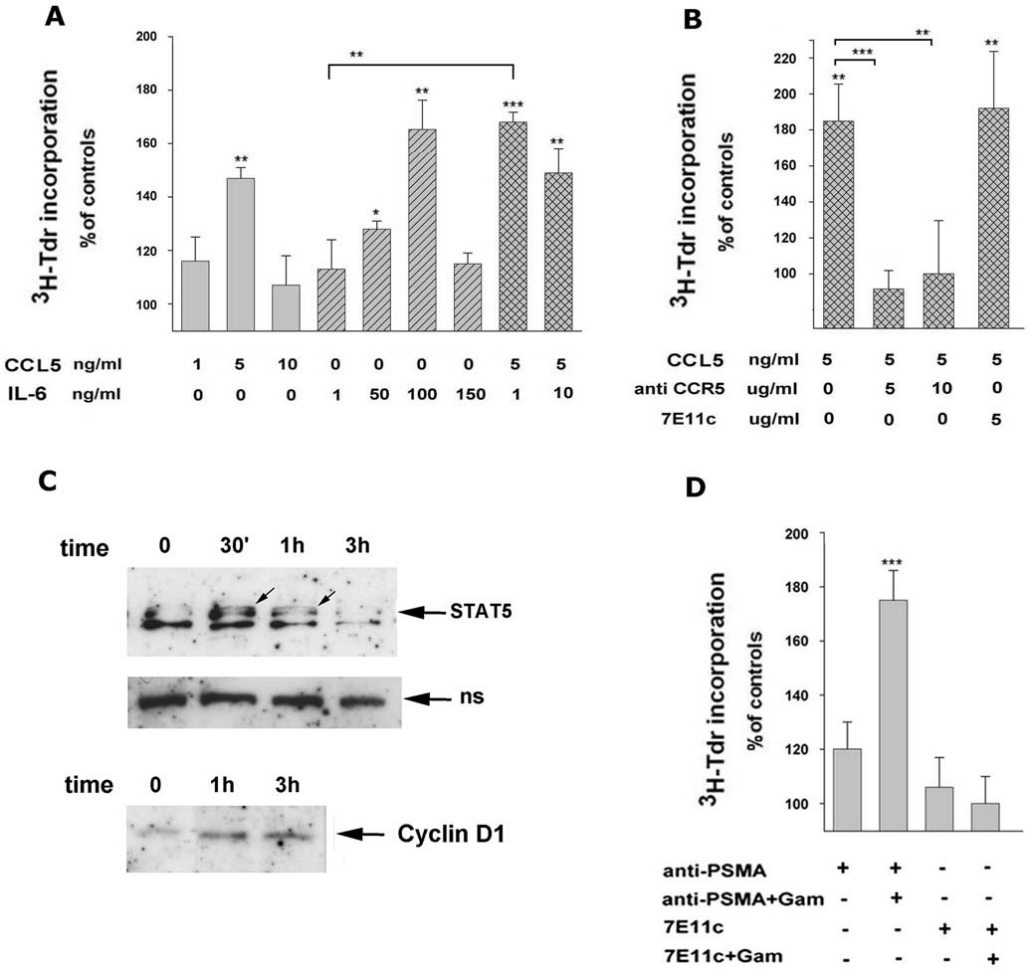
Effects of pro-proliferative stimuli on LNCaP cells. A) LNCaP proliferation induced by treatment with CCL5 and/or IL-6. ^3^H-TdR uptake of cells after 24 h of culture with CCL5 (grey bars), 72 h of culture with IL-6 (hatched bars) or 24 h of culture with combinations of CCL5 and IL-6 (crossed bars) at the indicated concentrations. B) ^3^H-TdR uptake of cells after 24 h culture with 5 ng/ml of CCL5 in the presence or in the absence of anti-CCR5 or 7E11c antibodies at the indicated concentrations; C) Western blot analysis of cell extracts (20 µg/lane) of LNCaP cells treated with CCL5 (5 ng/ml) and probed with anti-STAT 5 or with anti-cyclin D1 antibodies. Equal protein loading in all the lanes is confirmed by not specific bands shown in the middle gel. Arrows indicate phosphorylated STAT5 proteins. Western blotting results are representative of two independent experiments. D) ^3^H-TdR uptake of cells 72 h after binding or cross-linking of 7E11c or anti-PSMA antibodies. Values represent the mean±SEM of three independent experiments performed in triplicates. Stars on the bars indicate statistical significance as in Fig. 4.

As illustrated in Fig. 5 A, the rate of unlimited proliferation of LNCaP cells was increased by the addition of recombinant IL-6 or CCL5 in a dose-dependent manner. IL-6 exerted the maximal activity at 100 ng/ml (175±16, p<0.01) whereas CCL5 promoted a similar enhancement already at 5 ng/ml (146%±2 of increase, p<0.05), although only a subset (8–10%) of strongly positive CCR5 cells could be detected in our LNCaP cells population when FBS was lowered in culture to 1% (S. Grasso, personal observation). IL-6 and CCL5 appeared to act synergistically in inducing LNCaP cells proliferation, as demonstrated by the finding that the addition of as little as 1 ng/ml of IL-6 to the most effective dose of CCL5 raised the LNCaP cells proliferation up to values which were significantly higher than those of controls (168%±2.9, p<0.001), and, perhaps more importantly, greater than those observed in the presence of 5 ng/ml of CCL5 (p<001). As shown in Fig. 5 B the induction of proliferation induced by CCL5 was almost abrogated by blocking CCR5 with specific antibodies. No interference was instead observed in the presence of 7E11c antibody, used as isotype-matched control, thus putting into evidence a direct correlation between the burst of LNCaP cells proliferation and the CCL5 interaction with its receptor CCR5. It has been reported that the triggering of CCR5 by CCL5 is rapidly followed by JAK-STAT phosphorylation, more specifically by STAT1 and/or STAT3 and/or STAT5, depending on the cellular system [26]–[28]. It has been also reported that STAT5 phosphorylation regulates the expression of cyclin D1 in tumor cells including those of prostate [29], [30]. To investigate the downstream effects of CCR5 engagement in LNCaP cells, the cells were cultured in the presence of 5 ng/ml of CCL5 for the indicated times and then analysed as for the expression of STAT 5 and Cyclin D1 by immunoblotting with specific antibodies. As illustrated in Fig. 5 C phosphorylated STAT5 appeared as a doublet at 30 min. The doublet lowered its intensity at 1 h to return to the almost undetectable level observed in untreated cell at 3 h. Consistently, the amount of cyclin D1 was found more than doubled at 1 h and at 3 h, thus indicating that CCL5 can regulate LNCaP cells proliferation in a STAT 5-Cyclin D1 dependent way. Taken toghether, these results demonstrate that the antibody-mediated clustering of PSMA at the LNCaP cells surface activates signalling pathways able to directly or indirectly control the LNCaP cells growth. To confirm that the complex series of phenomena reported in the previous paragraphs led to LNCaP cells proliferation we decided to test if the cross-linking of PSMA would indeed induce the LNCaP cells proliferation. As shown in Fig. 5D LNCaP cells proliferation was found to be increased following PSMA cross-linking (175%±11, p<0.05), whereas the bindings of the sole anti-PSMA, of 7E11c or the cross-linking of 7E11c were all devoid of significant activity.

## Discussion

In this paper we show for the first time that the tumor biomarker PSMA, a multi-functional cell surface ectopeptidase [13], is endowed with an efficient signaling activity in prostate cancer cells. PSMA recruitment at the surface of LNCaP cells elicits a signaling wave involving small GTPases RAS, RAC1, and MAPK p38 and ERK1/2 activation. Consistent with the p38 and ERK1/2 phosphorylation, the NF-κB transcription factor was also activated [1] following PSMA stimulation and the minimal basal level of both IL-6 and CCL5 production was increased two-three fold. The discrete role of PSMA-mediated activation of p38 and ERK1/2 was examined under loss-of-function conditions secured by the blocking activity of kinase-specific chemical inhibitors, used at concentrations not affecting the adhesion of LNCaP cells nor the expression of PSMA at their surface (not shown). In this setting, p38 kinase exerted a wider and greater activity than ERK1/2 on both the basal and the PSMA-induced expression of IL-6 and CCL5. A possible explanation of the greater function of p38 may reside in the different roles played by p38 and ERK1/2 in the activation of NF-κB and NF-IL6, major transactivators of both IL-6 and CCL5 promoters [1]. We have previously reported that in normal human thymic epithelial cells p38 MAPK induced the post-transcriptional activation of both NF-κB and NF-IL6, whereas ERK1/2 was involved in the activation of NF-κB only. Consistent with the dual activity of p38, its inhibition with SB202190 almost abrogated the cytokine production, whereas ERK1/2 inhibition with PD98059 resulted in a lesser effect [24], [25]. The distinct functions of the two MAPKs may well be reproduced also in prostate cancer cells, as evidenced by our results with the inhibitors PD98059 and SB202190, thus suggesting that also in LNCaP cells p38 MAPK exerts a wider role than ERK1/2 on IL-6 and CCL5 transcription factors.

The membrane proximal events leading to RAS and RAC1 activation following PSMA recruitment have not been studied yet and full elucidation of these aspects will require further investigations.

Irrespective of the composition of the signaling platform used by PSMA in transducing external stimuli, our results provide evidence that PMSA can regulate the gene expression of IL-6 and CCL5 in the same LNCaP cells. These results were obtained following direct stimulation of the molecule under “in vitro” conditions closely resembling a physiological setting and without substantial cell manipulations. In addition, because biochemical studies and functional assays were performed with cell lysates and cell supernatants obtained from the same cell cultures, these results directly demonstrated that PSMA-elicited signals flowed efficiently from the membrane to the nucleus. The amount of IL-6 and CCL5 released in culture by the LNCaP cell line may appear lower than that produced by other cell lines. However, it has to be considered that a low concentration measured in cell supernatants can be dramatically high for a portion of the cell membrane and that the LNCaP cell line used by us represents only one of the multiple clones composing the prostate cancer cell population, thus emphasizing the importance that the synergic activity of IL-6 and CCL5 on LNCaP cells proliferation described by us may have in an “vivo” microenvironment. IL-6 has yielded conflicting results in prostate tumor cell lines including LNCaP. Cell proliferation, anti-apoptotic activity as well as growth arrest and neuroendocrine differentiation were all observed following treatment with IL-6 “in vitro” [12]. By contrast, the notion that the level of IL-6 and its receptor increase during prostate carcinogenesis “in vivo” is still unchallenged, so that IL-6 is nowadays considered a good candidate for targeted therapy in prostate cancer [12]. Differences in the cellular and experimental systems employed together with the complexity of the signaling pathway elicited by cytokines may possibly explain the divergence between the clinical and the “in vitro” experimental evidence.

Little is known about CCL5-CCR5 interaction in LNCaP cells, apart from the pro-proliferative activity previously reported by G. Vaday et al. [10] and confirmed also in the present paper. The novel finding presented herein is the ability of CCL5 to activate STAT 5 thereby regulating the expression of cyclin D1 and ultimately controlling the LNCaP cell cycle progression.

Overall, it appears that PSMA signaling may be involved at multiple stages of the LNCaP cell deregulation and possibly also of in vivo tumorigenesis, because 1) increased ERK1/2 activation correlates with increasing Gleason score and advanced tumor stage [31], and 2) increased activation of STAT 5 has been observed “in vivo” in 65% of human prostate cancers, being associated with high histological grade and being a predictor of early disease recurrence [29], [30], [32]. Moreover, STAT 5 inhibition induces cell death in human prostate cancer cells [33].

It could be hypothesized that following stimulation via PSMA the prostate tumor cells augment IL-6 and CCL5 production which are in turn used as growth factors in both autocrine and paracrine manner, triggering a cell proliferation/survival loop conferring resistance to apoptosis and an overall definite advantage to tumor cell populations. Within this context, the ability of PSMA to activate RAC1 may be highly relevant, inasmuch as RAC1 activation decreases the expression of E-cadherins thereby loosening intercellular adhesions and facilitating the cytoskeletal rearrangements required for mitosis [34]. If so, activated RAC1 could therefore favor the response of LNCaP cells to the mitogenic activity of IL-6 and CCL5 meanwhile participating to the induction of their expression.

These events may have a direct bearing also on tumor cell proliferation and survival “in vivo”. The prostate gland is composed mainly of stromal, epithelial, and neuroendocrine cells. The dynamic balance of cell proliferation, differentiation, and apoptosis maintains the cellular and tissue homeostasis. This balance is generated by a continuous cross-talk among these cell populations resulting from cell-cell contacts and the release of growth factors, chemokines and neuropeptides acting in a paracrine and autocrine manner [13]. Deregulation in this communication circuit may contribute to the derangement of one or more tissue component(s) of the prostate gland, possibly resulting in benign prostate hyperplasia or prostate carcinoma. The pathway components here described are already the targets of therapeutic interventions with pharmacologic or biotechnologic inhibitors in other types of epithelial tumors [21]. Therefore, also in prostate tumors the biochemical intermediates involved in NF-κB activation and augmented IL-6/CCL5 gene expression might represent adequate therapeutic targets aiming at interfering with one or more of the MAPKs activation pathway(s), thereby counteracting their effects on tumor cell proliferation and migration
