# Correction: The Prostate Specific Membrane Antigen Regulates the Expression of IL-6 and CCL5 in Prostate Tumour Cells by Activating the MAPK Pathways^1^

**DOI:** 10.1371/annotation/f290f38a-4f71-43ad-b988-9f8d5a7329fe

**Published:** 2009-03-31

**Authors:** Marco Colombatti, Silvia Grasso, Alessandra Porzia, Giulio Fracasso, Maria Teresa Scupoli, Sara Cingarlini, Ornella Poffe, Hassan Y. Naim, Martin Heine, Giuseppe Tridente, Fabrizio Mainiero, Dunia Ramarli

The following funding information was omitted. MC also received Fondazione Cariplo grant 2005-1166 (to S.Canevari). 

